# Comparison of Survival After Breast-Conserving Therapy vs Mastectomy Among Patients With or Without the *BRCA1/2* Variant in a Large Series of Unselected Chinese Patients With Breast Cancer

**DOI:** 10.1001/jamanetworkopen.2021.6259

**Published:** 2021-04-23

**Authors:** Qiting Wan, Liming Su, Tao Ouyang, Jinfeng Li, Tianfeng Wang, Zhaoqing Fan, Tie Fan, Benyao Lin, Yuntao Xie

**Affiliations:** 1 Breast Center, Key Laboratory of Carcinogenesis and Translational Research (Ministry of Education/Beijing), Peking University Cancer Hospital and Institute, Beijing, China

## Abstract

**Importance:**

Whether patients with breast cancer who carry a *BRCA1/2* variant can safely undergo breast-conserving therapy (BCT) remains controversial.

**Objective:**

To compare survival rates after BCT vs mastectomy in *BRCA1/2* variant carriers and noncarriers in a large series of unselected patients with breast cancer.

**Design, Setting, and Participants:**

In this cohort study, a large consecutive series of 8396 unselected patients with primary breast cancer underwent either BCT, mastectomy with radiotherapy, or mastectomy alone from October 1, 2003, to May 31, 2015, at the Breast Center of Peking University Cancer Hospital in China. All patients were assessed for *BRCA1/2* germline variant status. Statistical analysis was performed from May 1 to September 30, 2020.

**Main Outcomes and Measures:**

The primary outcomes were breast cancer–specific survival (BCSS) and overall survival (OS); secondary outcomes included recurrence-free survival, distant recurrence–free survival, and ipsilateral breast tumor recurrence.

**Results:**

Of these 8396 Chinese patients (8378 women [99.8% women]; mean [SD] age, 50.8 [11.4] years; 187 *BRCA1* carriers, 304 *BRCA2* carriers, and 7905 noncarriers), 3135 (37.3%) received BCT, 1511 (18.0%) received mastectomy with radiotherapy, and 3750 (44.7%) received mastectomy alone. After a median follow-up of 7.5 years (range, 0.3-16.6 years), both *BRCA1* and *BRCA2* variant carriers treated with BCT had similar rates of survival compared with those treated with mastectomy with radiotherapy (BCSS: hazard ratio [HR] for *BRCA1*, 0.58 [95% CI, 0.16-2.10]; *P* = .41; HR for *BRCA2*, 0.46 [95% CI, 0.15-1.41]; *P* = .17; OS: HR for *BRCA1*, 0.61 [95% CI, 0.18-2.12]; *P* = .44; HR for *BRCA2*, 0.72 [95% CI, 0.26-1.96]; *P* = .52) or mastectomy alone (BCSS: HR for *BRCA1*, 0.70 [95% CI, 0.22-2.20]; *P* = .54; HR for *BRCA2*, 0.59 [95% CI, 0.18-1.93]; *P* = .39; OS: HR for *BRCA1*, 0.77 [95% CI, 0.27-2.21]; *P* = .63; HR for *BRCA2*, 0.62 [95% CI, 0.22-1.73]; *P* = .37) after adjusting for clinicopathologic factors and adjuvant therapy. For noncarriers, patients receiving BCT had significantly better survival than those receiving mastectomy with radiotherapy (BCSS: HR, 0.45 [95% CI, 0.36-0.57]; *P* < .001; OS: HR, 0.46 [95% CI, 0.37-0.58]; *P* < .001) or mastectomy alone (BCSS: HR, 0.71 [95% CI, 0.57-0.89]; *P* = .003; OS: HR, 0.71 [95% CI, 0.58-0.87]; *P* < .001) in multivariable analyses.

**Conclusions and Relevance:**

This study suggests that *BRCA1/2* variant carriers treated with BCT have survival rates at least comparable to those treated with mastectomy with radiotherapy or mastectomy alone and that BCT could be an option for *BRCA1/2* variant carriers when the tumor is clinically appropriate for BCT.

## Key Points

**Question:**

Can patients with breast cancer who carry a *BRCA1/2* variant safely undergo breast-conserving therapy (BCT)?

**Findings:**

In this cohort study, 8396 patients with operable primary breast cancer (187 *BRCA1* carriers, 304 *BRCA2* carriers, and 7905 noncarriers) underwent BCT, mastectomy with radiotherapy, or mastectomy alone. In multivariable analyses, patients with both the *BRCA1* and *BRCA2* variants who were treated with BCT had a survival rate at least comparable to those treated with mastectomy with radiotherapy or mastectomy alone.

**Meaning:**

This study suggests that BCT may be an option for patients who carry a *BRCA1/2* variant when the tumor is clinically appropriate for this procedure.

## Teaser

This cohort study compares survival rates after breast-conserving therapy vs mastectomy in *BRCA1/2* variant carriers and noncarriers in a large series of unselected patients with breast cancer.

## Introduction

Early randomized clinical trials have demonstrated that breast-conserving therapy (BCT) with radiotherapy is equivalent to mastectomy in overall survival (OS) and breast cancer–specific survival (BCSS) among patients with early-stage breast cancer.^1,2,3,4,5^ Since then, BCT has been widely used for patients with early-stage breast cancer worldwide for more than 2 decades. Recent analyses based on population or registry studies involving many patients showed that BCT is associated with even better OS than mastectomy among patients with early-stage breast cancer.^6,7,8,9,10,11,12,13,14,15^ By contrast, it is still debated whether BCT could be safely used for *BRCA1* (OMIM 113705) and *BRCA2* (OMIM 600185) variant carriers with breast cancer. It is well documented that women carrying a germline variant in *BRCA1/2* genes not only have a high risk of breast cancer but are also at increased risk of developing a second primary breast cancer, particularly in the contralateral breast.^16,17,18,19,20,21,22,23,24,25,26^ Although the results are not fully in concordance, many studies showed that *BRCA1/2* variant carriers receiving BCT experienced a higher rate of ipsilateral breast cancer recurrence (especially second primary tumor) than did noncarriers.^17,18,23,25,26^ Therefore, the potential high risk of local recurrence after BCT and other unique features in *BRCA1/2* variant carriers with breast cancer raised concerns about whether BCT is associated with survival compared with mastectomy.

Several studies have addressed this issue and found no significant difference in survival rates between *BRCA1/2* variant carriers who underwent BCT and those who underwent mastectomy.^27,28,29,30^ These studies, in general, have been limited to a relatively small number of variant carriers, especially *BRCA2* variant carriers. In this study, therefore, we compared the survival rates between patients who underwent BCT and patients who underwent mastectomy with or without radiotherapy from a relatively large number of *BRCA1/2* variant carriers and noncarriers who were drawn from a large series of unselected patients with breast cancer.

## Methods

### Study Design

We conducted a retrospective cohort study to compare the survival rates of *BRCA1/2* variant carriers and noncarriers who underwent BCT, mastectomy with radiotherapy, or mastectomy alone at the Breast Center of Peking University Cancer Hospital from October 1, 2003, to May 31, 2015, in a large consecutive series of patients with operable primary invasive breast cancer who were unselected for age at diagnosis and family history of breast cancer. We also investigated the risk of recurrence in the ipsilateral breast and the risk of contralateral breast cancer in *BRCA1/2* variant carriers and noncarriers when they underwent different surgical procedures. Follow-up was censored on May 1, 2020. Written informed consent was obtained from all participants whose blood samples could be used for research, including genetic testing. We detected *BRCA1/2* germline variants for research purposes. Therefore, the patients and physicians were unaware of the *BRCA1/2* variant status when the patients were treated at the breast center. This study was approved by the research and ethics committee of Peking University Cancer Hospital. This study followed the Strengthening the Reporting of Observational Studies in Epidemiology (STROBE) reporting guideline.

### Patients and Procedures

A total of 10 269 consecutive patients with primary breast cancer were treated at the breast center, among whom 9822 patients had sufficient and qualified genomic DNA for *BRCA1/2* germline variant sequencing. The following patients were excluded from this study: patients with stage IV disease at diagnosis, follow-up time less than 3 months or loss of follow-up after surgery, distant metastasis within 3 months after primary diagnosis, noninvasive breast cancer, no operation was performed, patients receiving breast-conserving surgery who refused to receive radiotherapy, and whether radiotherapy was applied or not was unknown after surgery. The final sample for this retrospective study included 8396 Chinese patients with operable primary invasive breast cancer (stages I-III) (eFigure 1 in the Supplement).

Clear margins were required for BCT; a frozen section diagnosis was performed to judge whether the margins were clear. Patients who underwent BCT and those who underwent mastectomy who have a large tumor (ie, >5 cm) and/or 4 positive lymph nodes are referred for radiotherapy, whereas patients with 1 to 3 positive lymph nodes may receive radiotherapy if they have other high-risk factors. Patients may receive neoadjuvant or adjuvant chemotherapy or endocrine therapy depending on tumor clinicopathologic characteristics and clinical stage.

### *BRCA1/2* Variant

Germline DNA extracted from peripheral blood samples was tested for the *BRCA1/2* variant with multigene panel sequencing^31^ and/or Sanger sequencing^32^ or multiplex ligation–dependent probe amplification^33^ as described in previous studies. Only variants with definite pathogenicity were included. Of these 8396 patients, 187 (2.2%) carried a *BRCA1* variant, 304 (3.6%) carried a *BRCA2* variant, and 7905 (94.2%) were noncarriers.

### Statistical Analysis

Statistical analysis was performed from May 1 to September 30, 2020. The common clinicopathologic characteristics and the types of adjuvant therapy were compared among *BRCA1* variant carriers, *BRCA2* variant carriers, and noncarriers receiving different types of surgery. Continuous variables were compared using a *t* test, and categorical variables were compared using the Pearson χ^2^ test.

Follow-up started at the time of pathologic diagnosis from core needle biopsy or surgery. The primary outcomes included BCSS and OS, and the secondary outcomes were recurrence-free survival (RFS) and distant recurrence–free survival (DRFS). Breast cancer–specific survival was defined as the time from the date of diagnosis to death from breast cancer. Overall survival was calculated from the date of diagnosis to death from any cause. Recurrence-free survival was defined as the time from the date of pathologic diagnosis to the date of ipsilateral, regional, or distant recurrence or death from any cause but not including contralateral breast cancer. Distant recurrence–free survival was calculated from the date of diagnosis to distant recurrence or death from any cause. For patients who are alive, the latest date of follow-up was the last time they visited the physician or received a telephone call from the follow-up office of Peking University Cancer Hospital. Survival analyses were conducted using the Kaplan-Meier method, and the log-rank test was used to test differences between survival curves. Univariate and multivariable Cox proportional hazards regression models were used to assess the association between surgery procedures and survival. Multivariable adjustments were conducted for common clinicopathologic characteristics that are clinically relevant, including age at first breast cancer diagnosis; family history of breast and ovarian cancer; year of diagnosis; estrogen receptor, progesterone receptor, and ERBB2 status; nodal status; tumor size and grade; and adjuvant therapy.

Other secondary end points included ipsilateral breast tumor recurrence (IBTR) and contralateral breast cancer. Ipsilateral breast tumor recurrence was defined as a recurrence in the ipsilateral breast or chest wall no matter whether it was true tumor recurrence or a second primary tumor. Contralateral breast cancer was defined as developing invasive breast cancer or ductal carcinoma in situ in the contralateral breast at least 3 months after the primary breast cancer.

Tests were considered statistically significant at a 2-sided *P* < .05. All statistical analyses were completed in SPSS, version 23.0 software (SPSS Inc).

## Results

### Cohort Description

A total of 8396 patients (mean [SD] age, 50.8 [11.4] years) with primary invasive breast cancer (stages I-III), including 491 *BRCA1/2* variant carriers (5.8%) (187 *BRCA1* carriers [2.2%] and 304 *BRCA2* carriers [3.6%]) and 7905 noncarriers (94.2%), were included in the final analyses. The detailed clinicopathologic characteristics and treatment information are presented in Table 1.

**Table 1.** Clinicopathologic Characteristics of *BRCA1* and *BRCA2* Variant Carriers and Noncarriers in the Entire Cohort

Characteristic	No. (N = 8396)	No. (%)			*P* value		
		*BRCA1* carriers (n = 187 [2.2%])	*BRCA2* carriers (n =304 [3.6%])	Noncarriers (n = 7905 [94.2%])	*BRCA1* carriers vs noncarriers	*BRCA2* carriers vs noncarriers	*BRCA1* carriers vs *BRCA2* carriers
Surgery							
BCT	3135	73 (39.0)	106 (34.9)	2956 (37.4)	.79	.18	.25
Mastectomy plus RT	1511	30 (16.0)	67 (22.0)	1414 (17.9)			
Mastectomy alone	3750	84 (44.9)	131 (43.1)	3535 (44.7)			
Follow-up, y							
Mean (SD)	8.1 (3.2)	8.1 (3.5)	7.9 (3.2)	8.1 (3.2)	.76	.50	.52
Median (range)	7.5 (0.3-16.6)	7.7 (0.8-15.6)	7.3 (0.4-16.0)	7.5 (0.3-16.6)			
Age, y							
Mean (SD)	50.8 (11.4)	44.8 (10.0)	47.6 (10.4)	51.0 (11.4)	<.001	<.001	.004
Median (range)	50 (19-90)	43 (27-81)	47 (21-75)	51 (19-90)			
≤45	2913	110 (58.8)	130 (42.8)	2673 (33.8)	<.001	.001	.001
>45	5483	77 (41.2)	174 (57.2)	5232 (66.2)			
Year of diagnosis							
2003-2009	3480	87 (46.5)	111 (36.5)	3282 (41.5)	.17	.08	.03
2010-2015	4916	100 (53.5)	193 (63.5)	4623 (58.5)			
Family history of any cancer							
Yes	2632	112 (59.9)	166 (54.6)	2354 (29.8)	<.001	<.001	<.001
No	5764	75 (40.1)	138 (45.4)	5551 (70.2)			
Family history of breast and/or ovarian cancer							
Yes	799	71 (38.0)	100 (32.9)	628 (7.9)	<.001	<.001	.25
No	7597	116 (62.0)	204 (67.1)	7277 (92.1)			
ER status^a^							
Positive	6071	59 (31.7)	242 (79.6)	5770 (73.0)	<.001	.01	<.001
Negative	2295	127 (68.3)	62 (20.4)	2106 (26.6)			
Unknown	30	1	0	29			
PR status^a^							
Positive	5475	55 (29.6)	224 (73.7)	5196 (65.7)	<.001	.006	<.001
Negative	2886	131 (70.4)	80 (26.3)	2675 (33.8)			
Unknown	35	1	0	34			
ERBB2 status^a^							
Positive	1986	13 (7.0)	35 (11.5)	1938 (24.6)	<.001	<.001	.10
Negative	6369	173 (93.0)	269 (88.5)	5927 (75.4)			
Unknown	41	1	0	40			
Molecular type^a^							
ER positive, PR positive, and ERBB2 negative	5197	58 (31.2)	225 (74.0)	4914 (62.5)	<.001	<.001	<.001
ER negative, PR negative, and ERBB2 negative	1171	115 (61.8)	44 (14.5)	1012 (12.9)			
ERBB2 positive	1986	13 (7.0)	35 (11.5)	1938 (24.6)			
Unknown	42	1	0	41			
Stage^a^							
I	2490	60 (32.4)	83 (27.8)	2347 (30.5)	.20	.22	.05
II	4664	110 (59.5)	169 (56.5)	4385 (57.0)			
III	1027	15 (8.1)	47 (15.7)	965 (12.5)			
Unknown	215	2	5	208			
Grade^a^							
I and II	6170	104 (61.2)	221 (81.5)	5845 (84.8)	<.001	.15	<.001
III	1165	66 (38.8)	50 (18.5)	1049 (15.2)			
Unknown	1061	17	33	1011			
Nodal status^a^							
0	5200	131 (70.1)	174 (57.8)	4895 (62.5)	.05	.10	.005
1-3	2182	44 (23.5)	82 (27.2)	2056 (26.3)			
≥4	937	12 (6.4)	45 (15.0)	880 (11.2)			
Unknown	77	0	3	74			
Tumor size, cm^a,b^							
≤2	3647	74 (40.4)	137 (45.5)	3436 (44.5)	.29	.84	.55
>2 to 3	2626	67 (36.6)	93 (30.9)	2466 (31.9)			
>3 to 5	1544	30 (16.4)	54 (17.9)	1460 (18.9)			
>5	393	12 (6.6)	17 (5.6)	364 (4.7)			
Unknown	186	4	3	179			
Adjuvant therapy							
Chemotherapy	1972	115 (61.5)	60 (19.7)	1797 (22.7)	<.001	.003	<.001
Endocrine therapy	2050	11 (5.9)	59 (19.4)	1980 (25.0)			
Chemotherapy and endocrine therapy	4073	58 (31.0)	179 (58.9)	3836 (48.5)			
None	301	3 (1.6)	6 (2.0)	292 (3.7)			

Abbreviations: BCT, breast-conserving therapy; ER, estrogen receptor; PR, progesterone receptor; RT, radiotherapy.

^a^ Percentages calculated without unknown numbers included.

^b^ Tumor size is a continuous variable, so size categories overlap.

A total of 3135 patients (37.3%) received BCT, 1511 (18.0%) received mastectomy with radiotherapy, and 3750 (44.7%) received mastectomy alone. The percentages of patients who underwent BCT, mastectomy with radiotherapy, and mastectomy alone were similar among the *BRCA1* variant carriers, *BRCA2* variant carriers, and noncarriers (Table 1).

### Comparison of Survival Among *BRCA1* and *BRCA2* Variant Carriers and Noncarriers in the Entire Cohort

**Figure 1.**
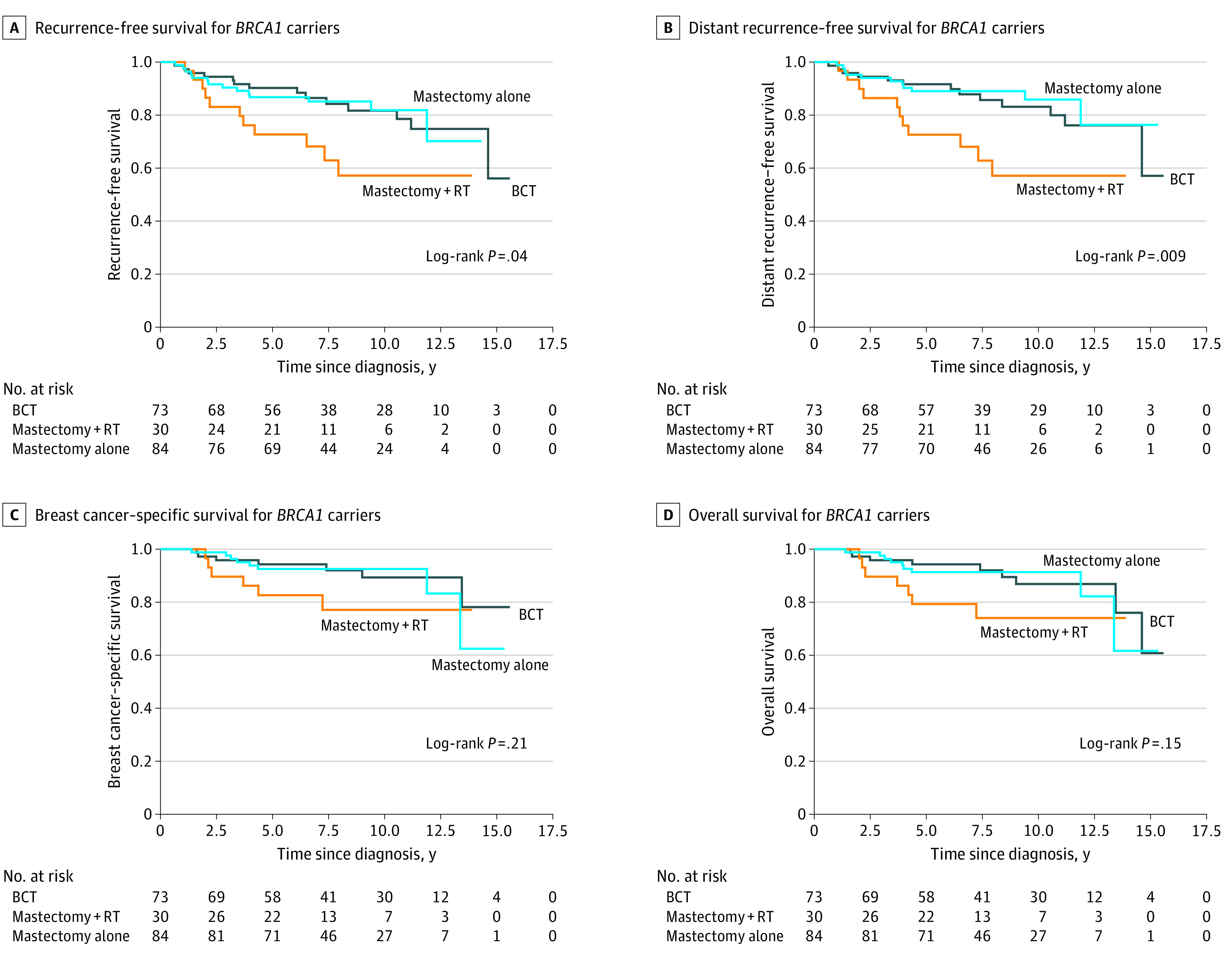
Survival of *BRCA1* Variant Carriers Receiving Breast-Conserving Therapy (BCT), Mastectomy With Radiotherapy (RT), or Mastectomy Alone

After a median follow-up of 7.5 years (range, 0.3-16.6 years) in the entire cohort, there were no significant differences in BCSS among *BRCA1* variant carriers (88.8%), *BRCA2* variant carriers (92.1%), and noncarriers (92.4%; log-rank, *P* = .22; eFigure 2 in the Supplement). Among the 187 *BRCA1* variant carriers, 73 (39.0%) underwent BCT, 30 (16.0%) underwent mastectomy with radiotherapy, and 84 (44.9%) underwent mastectomy alone; the clinicopathologic characteristics of the 3 groups are presented in eTable 1 in the Supplement. The Kaplan-Meier survival curves of RFS, DRFS, BCSS, and OS in the 3 treatment subgroups of *BRCA1* variant carriers are presented in Figure 1. Multivariable analyses showed that *BRCA1* variant carriers treated with BCT had similar survival compared with those treated with mastectomy with radiotherapy (BCSS: hazard ratio [HR], 0.58 [95% CI, 0.16-2.10]; *P* = .41; OS: HR, 0.61 [95% CI, 0.18-2.12]; *P* = .44) or mastectomy alone (BCSS: HR, 0.70 [95% CI, 0.22-2.20]; *P* = .54; OS: HR, 0.77 [95% CI, 0.27-2.21]; *P* = .63) after adjusting for age at diagnosis, family history of breast and ovarian cancer, year of diagnosis, tumor size, tumor grade, lymph node, estrogen receptor, progesterone receptor, ERBB2, and adjuvant therapy (Table 2). The detailed data of multivariable analyses regarding RFS and DRFS among *BRCA1* variant carriers are presented in Table 2.

**Table 2.** Univariate and Multivariable Analyses of Survival for *BRCA1* and *BRCA2* Variant Carriers and Noncarriers Receiving BCT, Mastectomy With RT, or Mastectomy Alone

Survival, Surgery	Univariate analysis		Multivariable analysis^a^	
	HR (95% CI)	*P* value	HR (95% CI)	*P* value
*BRCA1* carriers				
RFS				
BCT vs mastectomy plus RT	0.41 (0.18-0.92)	.03	0.55 (0.21-1.44)	.23
BCT vs mastectomy alone	0.97 (0.45-2.07)	.94	1.04 (0.46-2.35)	.93
DRFS				
BCT vs mastectomy plus RT	0.38 (0.17-0.86)	.02	0.59 (0.22-1.61)	.31
BCT vs mastectomy alone	1.20 (0.53-2.70)	.67	1.20 (0.50-2.89)	.68
BCSS				
BCT vs mastectomy plus RT	0.43 (0.14-1.27)	.13	0.58 (0.16-2.10)	.41
BCT vs mastectomy alone	0.81 (0.29-2.28)	.69	0.70 (0.22-2.20)	.54
OS				
BCT vs mastectomy plus RT	0.42 (0.15-1.15)	.09	0.61 (0.18-2.12)	.44
BCT vs mastectomy alone	0.87 (0.34-2.26)	.78	0.77 (0.27-2.21)	.63
*BRCA2* carriers				
RFS				
BCT vs mastectomy plus RT	0.42 (0.22-0.80)	.009	0.71 (0.34-1.49)	.37
BCT vs mastectomy alone	0.96 (0.49-1.86)	.90	0.73 (0.36-1.48)	.38
DRFS				
BCT vs mastectomy plus RT	0.39 (0.20-0.76)	.006	0.66 (0.31-1.44)	.30
BCT vs mastectomy alone	1.06 (0.51-2.19)	.88	0.83 (0.39-1.79)	.64
BCSS				
BCT vs mastectomy plus RT	0.31 (0.11-0.83)	.02	0.46 (0.15-1.41)	.17
BCT vs mastectomy alone	0.96 (0.32-2.87)	.95	0.59 (0.18-1.93)	.39
OS				
BCT vs mastectomy plus RT	0.37 (0.15-0.92)	.03	0.72 (0.26-1.96)	.52
BCT vs mastectomy alone	0.99 (0.38-2.56)	.98	0.62 (0.22-1.73)	.37
Noncarriers				
RFS				
BCT vs mastectomy plus RT	0.35 (0.30-0.40)	<.001	0.63 (0.54-0.74)	<.001
BCT vs mastectomy alone	0.87 (0.76-1.00)	.05	0.84 (0.73-0.97)	.02
DRFS				
BCT vs mastectomy plus RT	0.27 (0.24-0.32)	<.001	0.49 (0.42-0.59)	<.001
BCT vs mastectomy alone	0.77 (0.66-0.90)	.001	0.75 (0.64-0.88)	<.001
BCSS				
BCT vs mastectomy plus RT	0.22 (0.18-0.28)	<.001	0.45 (0.36-0.57)	<.001
BCT vs mastectomy alone	0.75 (0.61-0.93)	.009	0.71 (0.57-0.89)	.003
OS				
BCT vs mastectomy plus RT	0.25 (0.21-0.31)	<.001	0.46 (0.37-0.58)	<.001
BCT vs mastectomy alone	0.70 (0.58-0.85)	<.001	0.71 (0.58-0.87)	<.001

Abbreviations: BCSS, breast cancer–specific survival; BCT, breast-conserving therapy; DRFS, distant recurrence–free survival; HR, hazard ratio; OS, overall survival; RFS, recurrence-free survival; RT, radiotherapy.

^a^ Hazard ratio adjusted for clinicopathologic characteristics and treatment factors including age at first breast cancer diagnosis (≤45 vs >45 years), family history of breast and ovarian cancer (with vs without), year of diagnosis (2003-2009 vs 2010-2015), estrogen receptor status, progesterone receptor status, ERBB2 status, lymph node status (positive vs negative), tumor size (>3 vs ≤3 cm), grade (III vs I and II; unknown vs I and II), and adjuvant therapy (chemotherapy vs endocrine therapy or no therapy).

**Figure 2.**
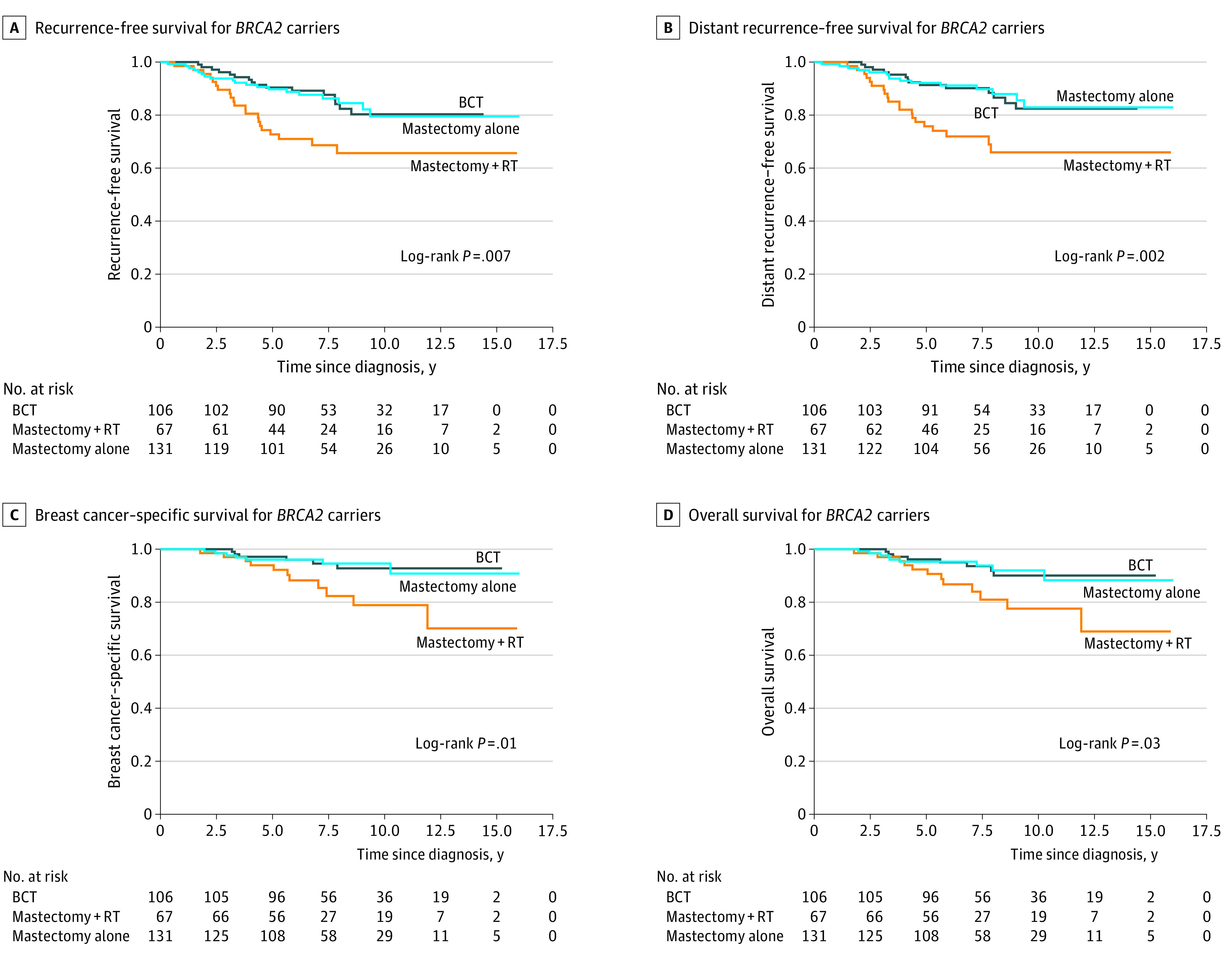
Survival of *BRCA2* Variant Carriers Receiving Breast-Conserving Therapy (BCT), Mastectomy With Radiotherapy (RT), or Mastectomy Alone

Among the 304 *BRCA2* variant carriers, 106 (34.9%) underwent BCT, 67 (22.0%) underwent mastectomy with radiotherapy, and 131 (43.1%) underwent mastectomy alone; the clinicopathologic characteristics of the 3 groups are presented in eTable 2 in the Supplement. The Kaplan-Meier survival curves of RFS, DRFS, BCSS, and OS in the 3 treatment subgroups of *BRCA2* variant carriers are presented in Figure 2. Multivariable analyses showed that *BRCA2* variant carriers treated with BCT had similar survival compared with those treated with mastectomy with radiotherapy (BCSS: HR, 0.46 [95% CI, 0.15-1.41]; *P* = .17; OS: HR, 0.72 [95% CI, 0.26-1.96]; *P* = .52) or mastectomy alone (BCSS: HR, 0.59 [95% CI, 0.18-1.93]; *P* = .39; OS: HR, 0.62 [95% CI, 0.22-1.73]; *P* = .37) (Table 2). The details of RFS and DRFS are presented in Table 2.

**Figure 3.**
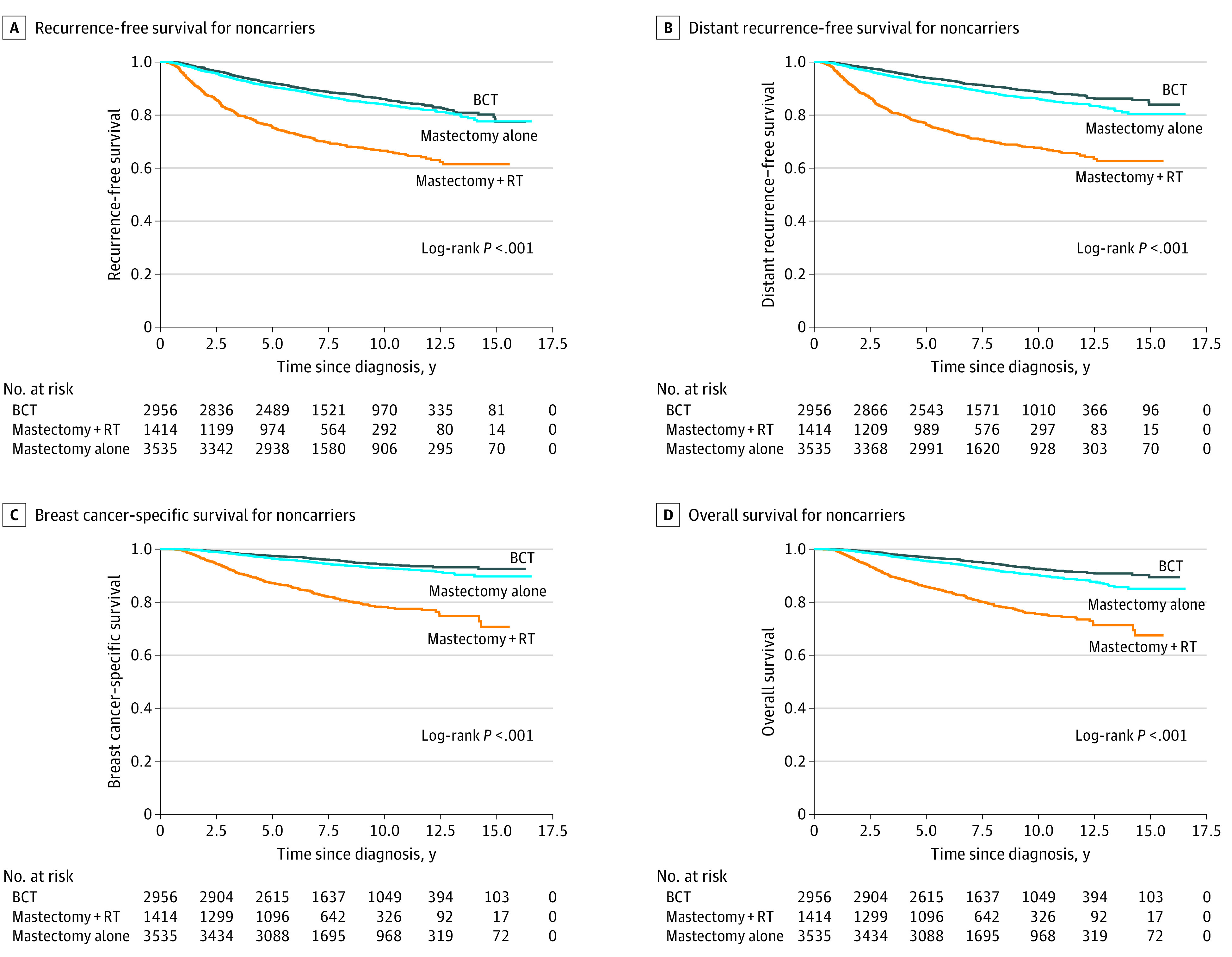
Survival of Noncarriers Receiving Breast-Conserving Therapy (BCT), Mastectomy With Radiotherapy (RT), or Mastectomy Alone

Among the 7905 noncarriers, 2956 (37.4%) underwent BCT, 1414 (17.9%) underwent mastectomy with radiotherapy, and 3535 (44.7%) underwent mastectomy alone; the clinicopathologic characteristics of the 3 groups are presented in eTable 3 in the Supplement. The Kaplan-Meier survival curves of RFS, DRFS, BCSS, and OS in the 3 treatment subgroups of noncarriers are presented in Figure 3. Noncarriers receiving BCT had a significantly better survival than those receiving mastectomy with radiotherapy (BCSS: HR, 0.45 [95% CI, 0.36-0.57]; *P* < .001; OS: HR, 0.46 [95% CI, 0.37-0.58]; *P* < .001) or mastectomy alone (BCSS; HR, 0.71 [95% CI, 0.57-0.89]; *P* = .003; OS: HR, 0.71 [95% CI, 0.58-0.87]; *P* < .001) in multivariable analyses (Table 2). The details of RFS and DRFS are presented in Table 2.

### Comparison of Survival Among *BRCA1* and *BRCA2* Variant Carriers and Noncarriers With Early-Stage Disease

Patients with early-stage disease (ie, stages I and II) may be suitable for either BCT or mastectomy. Therefore, in this cohort, we compared the survival rates between patients with stage I and II disease (T3N0M0 excluded) who underwent BCT and those who underwent mastectomy with or without radiotherapy. Multivariable analyses still showed that *BRCA1/2* variant carriers treated with BCT had a similar survival to those treated with mastectomy with radiotherapy (BCSS: HR for *BRCA1*, 1.31 [95% CI, 0.12-14.49]; *P* = .83; HR for *BRCA2*, 0.76 [95% CI, 0.13-4.50]; *P* = .77; OS: HR for *BRCA1*, 1.31 [95% CI, 0.13-13.33]; *P* = .82; HR for *BRCA2*, 0.98 [95% CI, 0.18-5.21]; *P* = .98) or mastectomy alone (BCSS: HR for *BRCA1*, 1.12 [95% CI, 0.30-4.22]; *P* = .87; HR for *BRCA2*, 0.90 [95% CI, 0.23-3.60]; *P* = .89; OS: HR for *BRCA1*, 1.29 [95% CI, 0.39-4.24]; *P* = .68; HR for *BRCA2*, 0.60 [95% CI, 0.16-2.20]; *P* = .44) (eTable 4 in the Supplement). Regarding noncarriers, we found that those who underwent BCT had significantly better survival than those who underwent mastectomy with radiotherapy (BCSS: HR, 0.56 [95% CI, 0.40-0.79]; *P* = .001; OS: HR, 0.58 [95% CI, 0.43-0.80]; *P* = .001) and those who underwent mastectomy alone (BCSS: HR, 0.69 [95% CI, 0.54-0.90]; *P* = .006; OS: HR, 0.68 [95% CI, 0.54-0.85]; *P* = .001) in multivariable analyses (eTable 4 in the Supplement).

### IBTR and Contralateral Breast Cancer

The rates of IBTR were 2.1% for *BRCA1* variant carriers (4 of 187), 4.6% for *BRCA2* variant carriers (14 of 304), and 3.1% for noncarriers (249 of 7905) in the entire cohort (eTable 5 in the Supplement). The IBTR rates among *BRCA1* variant carriers were 1.4% for BCT (1 of 73), 0.0% for mastectomy with radiotherapy (0 of 30), and 3.6% for mastectomy alone (3 of 84). The IBTR rates among *BRCA2* variant carriers were 7.5% for BCT (8 of 106), 1.5% for mastectomy with radiotherapy (1 of 67), and 3.8% for mastectomy alone (5 of 131). The IBTR rates among noncarriers were 3.9% for BCT (115 of 2956), 2.7% for mastectomy with radiotherapy (38 of 1414), and 2.7% for mastectomy alone (96 of 3535) (eTable 5 in the Supplement). *BRCA2* variant carriers who underwent BCT showed a nonsignificantly higher IBTR rate than *BRCA1* variant carriers and noncarriers who underwent BCT (odds ratio, 2.02 [95% CI, 0.96-4.25]; *P* = .07; eTable 6 in the Supplement).

The rates of contralateral breast cancer among *BRCA1* and *BRCA2* variant carriers were significantly higher than that among noncarriers (*BRCA1* vs noncarriers, 13.9% [26 of 187] vs 2.5% [198 of 7905]; *P* < .001; *BRCA2* vs noncarriers, 13.5% [41 of 304] vs 2.5% [198 of 7905]; *P* < .001) (eTable 7 in the Supplement). The rates of contralateral breast cancer among *BRCA1* variant carriers were 13.7% for those receiving BCT (10 of 73), 20.0% for those receiving mastectomy with radiotherapy (6 of 30), and 11.9% for those receiving mastectomy alone (10 of 84), whereas the rate of contralateral breast cancer was significantly higher among *BRCA2* variant carriers treated with mastectomy alone than among those treated with BCT (19.8% [26 of 131] vs 6.6% [7 of 106]; *P* = .003) and was nonsignificantly higher than among those treated with mastectomy with radiotherapy (19.8% [26 of 131] vs 11.9% [8 of 67]; *P* = .16).

## Discussion

In this cohort study of a large, consecutive series of 8396 Chinese patients with primary invasive breast cancer in a single center, we investigated whether *BRCA1/2* variant carriers could safely undergo BCT when the breast tumor was suitable for this procedure. We found that *BRCA1/2* variant carriers receiving BCT exhibited survival rates at least comparable to that of patients receiving mastectomy with or without radiotherapy after adjusting for clinicopathologic characteristics and adjuvant therapy. As expected, BCT was associated with significantly better survival than mastectomy with or without radiotherapy among noncarriers after adjusting for all confounding factors.

In this large, consecutive series of unselected patients with breast cancer, all of the patients and physicians were not aware of the *BRCA1/2* variant status when they selected BCT or mastectomy, largely depending on the tumor characteristics per se and the preferences of patients and physicians. Therefore, the percentage of patients who underwent BCT was similar throughout the subgroups (39.0% of *BRCA1* carriers, 34.9% of *BRCA2* carriers, and 37.4% of noncarriers). In addition, the *BRCA1/2* variant status was assessed for all of the participants. All of these features largely decreased selection bias. Therefore, our data represent the real world of clinical practice. Several previous studies also compared the survival rates of *BRCA1/2* variant carriers who underwent BCT or mastectomy and found no difference in BCSS and OS between BCT and mastectomy.^27,28,29,30^ However, the sample size of some studies was relatively small, particularly for *BRCA2* variant carriers.^28,29,30^ Therefore, these studies could not comprehensively analyze the survival of the *BRCA2* subgroup, especially for multivariable analyses.^27,28,29,30^ Our present study comprised a relatively large number of *BRCA1/2* variant carriers to date, particularly *BRCA2* variant carriers (304 cases). These advantages enabled us to analyze survival among the *BRCA1* and *BRCA2* subgroups in univariate and multivariable analyses. The results of our present study in combination with previous studies suggest that the survival rate among *BRCA1/2* variant carriers who underwent BCT and the survival rate among *BRCA1/2* variant carriers who underwent mastectomy were comparable.

On the other hand, we also noted that patients who received mastectomy with radiotherapy usually had a large tumor or more positive lymph nodes involved compared with those who received BCT or mastectomy alone. In the univariate analyses, we found that BCT showed significantly favorable survival compared with mastectomy with radiotherapy among *BRCA1/2* variant carriers and noncarriers, but these differences in *BRCA1/2* variant carriers did not reach significance in multivariable analyses. The main reason for these differences not reaching significance was the relatively small sample size of *BRCA1/2* variant carriers when stratified by 3 subgroups, particularly for *BRCA1* variant carriers. In contrast, BCT showed significantly favorable survival compared with mastectomy with radiotherapy among noncarriers after adjusting for clinicopathologic factors and adjuvant therapy.

We further performed an analysis among patients with clinical stage I and II disease (T3N0M0 excluded) who might be either suitable for BCT or mastectomy. We found that BCT showed a survival rate similar to mastectomy with or without radiotherapy among *BRCA1/2* variant carriers in this subset of patients.

In this study, the IBTR rates were relatively low among the groups in the entire cohort, and there was no significant difference in the *BRCA1* variant carrier, *BRCA2* variant carrier, and noncarrier groups among the patients who underwent BCT, although *BRCA2* variant carriers exhibited a nonsignificantly higher rate than noncarriers (7.5% vs 3.9%). These findings are in line with those of previous studies^19,20,21,22,24,26,34^ and indicate that the rates of IBTR were reasonably low among *BRCA1/2* variant carriers treated with BCT.

In contrast to IBTR, the rate of contralateral breast cancer was significantly higher among *BRCA1/2* variant carriers than among noncarriers regardless of BCT or mastectomy. The high risk of contralateral breast cancer may outweigh the benefits associated with BCT for *BRCA1/2* variant carriers. Clinicians may discuss this issue with *BRCA1/2* variant carriers who may face a high risk of contralateral breast cancer regardless of BCT or mastectomy. Therefore, a mastectomy and a contralateral prophylactic mastectomy with or without immediate reconstruction is an alternative option for *BRCA1/2* variant carriers.

### Limitations and Strengths

There are 2 limitations in our study. First, this was a retrospective study, and patients were not randomly assigned to treatment groups. Second, although the size of the entire cohort was large and the number of *BRCA1/2* variant carriers was relatively large, the number of variant carriers (ie, *BRCA1*) in the subgroups was relatively small. Thus, caution should be taken when estimating the rate of IBTR amongr *BRCA1/2* variant carriers. This study also has some strengths, including the relatively large number of *BRCA1/2* variant carriers and the comprehensive information on clinicopathologic characteristics and adjuvant therapy.

## Conclusions

In this study, we found that *BRCA1/2* variant carriers treated with BCT have a survival rate comparable to that of those treated with mastectomy with radiotherapy or mastectomy alone after adjusting for clinicopathologic factors and adjuvant therapy in this large, consecutive series of unselected patients with breast cancer. We also demonstrated that, with regard to survival rates, BCT is significantly superior to mastectomy with radiotherapy or mastectomy alone among noncarriers. Nevertheless, our present study clearly shows that BCT could be an option for *BRCA1/2* variant carriers when the breast tumor is clinically appropriate for the procedure.

## Notes

Supplement. **eTable 1.** Clinicopathologic Characteristics for *BRCA1* Variant Carriers Receiving Breast-Conserving Therapy, Mastectomy With Radiotherapy, or Mastectomy Alone **eTable 2.** Clinicopathologic Characteristics for *BRCA2* Variant Carriers Receiving Breast-Conserving Therapy, Mastectomy With Radiotherapy, or Mastectomy Alone **eTable 3.** Clinicopathologic Characteristics for Noncarriers Receiving Breast-Conserving Therapy, Mastectomy With Radiotherapy, or Mastectomy Alone **eTable 4.** Comparison of Survival Between Breast-Conserving Therapy, Mastectomy With Radiotherapy, and Mastectomy Alone in *BRCA1* and *BRCA2* Variant Carriers, and Noncarriers Among the Patients with Early-Stage Disease (T3N0M0 Excluded) **eTable 5.** Risk of Ipsilateral Breast Tumor Recurrence for Patients Receiving Different Type of Surgery Within *BRCA1* and *BRCA2* Variant Carriers, and Noncarriers **eTable 6.** Risk of Ipsilateral Breast Tumor Recurrence for *BRCA1* and *BRCA2* Variant Carriers, and Noncarriers in Subgroups of Different Type of Surgery **eTable 7.** Risk of Contralateral Breast Cancer for Patients Receiving Different Type of Surgery Within *BRCA1* and *BRCA2* Variant Carriers, and Noncarriers **eFigure 1.** Flow Diagram of the Patients Included in This Cohort **eFigure 2.** Survival Between *BRCA1* and *BRCA2* Variant Carriers, and Noncarriers in This Cohort
