# Supplementary material for: Comparison of Survival After Breast-Conserving Therapy vs Mastectomy Among Patients With or Without the BRCA1/2 Variant in a Large Series of Unselected Chinese Patients With Breast Cancer
Source: JAMA Netw Open. 2021 Apr 23;4(4):e216259. doi: 10.1001/jamanetworkopen.2021.6259 (PMC8065382; doi:10.1001/jamanetworkopen.2021.6259)
Supplement: Supplement. — eTable 1. Clinicopathologic Characteristics for BRCA1 Variant Carriers Receiving Breast-Conserving Therapy, Mastectomy With Radiotherapy, or Mastectomy Alone eTable 2. Clinicopathologic Characteristics for BRCA2 Variant Carriers Receiving Breast-Conserving Therapy, Mastectomy With Radiotherapy, or Mastectomy Alone eTable 3. Clinicopathologic Characteristics for Noncarriers Receiving Breast-Conserving Therapy, Mastectomy With Radiotherapy, or Mastectomy Alone eTable 4. Comparison of Survival Between Breast-Conserving Therapy, Mastectomy With Radiotherapy, and Mastectomy Alone in BRCA1 and BRCA2 Variant Carriers, and Noncarriers Among the Patients with Early-Stage Disease (T3N0M0 Excluded) eTable 5. Risk of Ipsilateral Breast Tumor Recurrence for Patients Receiving Different Type of Surgery Within BRCA1 and BRCA2 Variant Carriers, and Noncarriers eTable 6. Risk of Ipsilateral Breast Tumor Recurrence for BRCA1 and BRCA2 Variant Carriers, and Noncarriers in Subgroups of Different Type of Surgery eTable 7. Risk of Contralateral Breast Cancer for Patients Receiving Different Type of Surgery Within BRCA1 and BRCA2 Variant Carriers, and Noncarriers eFigure 1. Flow Diagram of the Patients Included in This Cohort eFigure 2. Survival Between BRCA1 and BRCA2 Variant Carriers, and Noncarriers in This Cohort [file jamanetwopen-e216259-s001.pdf]

Supplementary Online Content

Wan Q, Su L, Ouyang T, et al. Comparison of survival after breast-conserving therapy vs mastectomy among patients with or without the *BRCA1/2* variant in a large series of unselected Chinese patients with breast cancer. *JAMA Netw Open.* 2021;4(4):e216259. doi:10.1001/jamanetworkopen.2021.6259

eTable 1. Clinicopathologic Characteristics for *BRCA1* Variant Carriers Receiving Breast-Conserving Therapy, Mastectomy With Radiotherapy, or Mastectomy Alone

eTable 2. Clinicopathologic Characteristics for *BRCA2* Variant Carriers Receiving Breast-Conserving Therapy, Mastectomy With Radiotherapy, or Mastectomy Alone

eTable 3. Clinicopathologic Characteristics for Noncarriers Receiving Breast-Conserving Therapy, Mastectomy With Radiotherapy, or Mastectomy Alone

eTable 4. Comparison of Survival Between Breast-Conserving Therapy, Mastectomy With Radiotherapy, and Mastectomy Alone in *BRCA1* and *BRCA2* Variant Carriers, and Noncarriers Among the Patients with Early-Stage Disease (T3N0M0 Excluded)

eTable 5. Risk of Ipsilateral Breast Tumor Recurrence for Patients Receiving Different Type of Surgery Within *BRCA1* and *BRCA2* Variant Carriers, and Noncarriers

eTable 6. Risk of Ipsilateral Breast Tumor Recurrence for *BRCA1* and *BRCA2* Variant Carriers, and Noncarriers in Subgroups of Different Type of Surgery

eTable 7. Risk of Contralateral Breast Cancer for Patients Receiving Different Type of Surgery Within *BRCA1* and *BRCA2* Variant Carriers, and Noncarriers

eFigure 1. Flow Diagram of the Patients Included in This Cohort

eFigure 2. Survival between *BRCA1* and *BRCA2* Variant Carriers, and Noncarriers in This Cohort

This supplementary material has been provided by the authors to give readers additional information about their work.

© 2021 Wan Q et al. *JAMA Network Open.*

eTable 1. Clinicopathologic Characteristics for *BRCA1* Variant Carriers Receiving Breast-Conserving Therapy, Mastectomy With Radiotherapy, or Mastectomy Alone

**Characteristics**	**No.**	**BCT,**	**Mastectomy plus RT,**	**Mastectomy alone,**	**P[1]**	**P[2]**	**P[3]**
		**N (%)**	**N (%)**	**N (%)**			
Total	187	73 (39.0)	30 (16.0)	84 (44.9)			
Follow-up(years)							
Mean(±SD)	8.1±3.5	8.6±3.8	7.4±3.5	8.0±3.1	0.15	0.35	0.34
Median	7.7	8.0	7.0	7.7			
Range	0.8-15.6	0.8-15.6	1.9-13.9	1.2-15.3			
Age (years)							
Mean(±SD)	44.8±10.0	43.4±9.3	44.8±12.0	46.0±9.9	0.55	0.59	0.08
Median	43	42	42	45			
Range	27-81	27-68	28-81	28-73			
≤45	110	49 (67.1)	17 (56.7)	44 (52.4)	0.32	0.69	0.06
>45	77	24 (32.9)	13 (43.3)	40 (47.6)			
Year of diagnosis							
2003-2009	87	41 (56.2)	14 (46.7)	32 (38.1)	0.38	0.41	0.02
2010-2015	100	32 (43.8)	16 (53.3)	52 (61.9)			
Family history of any cancer							
Yes	112	54 (74.0)	13 (43.3)	45 (53.6)	0.003	0.34	0.008
No	75	19 (26.0)	17 (56.7)	39 (46.4)			
Family history of BCOC							
Yes	71	36 (49.3)	9 (30.0)	26 (31.0)	0.07	0.92	0.02
No	116	37 (50.7)	21 (70.0)	58 (69.0)			

© 2021 Wan Q et al. *JAMA Network Open.*

ER status[a]									
Positive	59	25 (34.2)		12 (41.4)		22 (26.2)	0.50	0.12	0.27
Negative	127	48 (65.8)		17 (58.6)		62 (73.8)			
Unknown	1	0		1		0			
PR status[a]									
Positive	55	18 (24.7)		13 (44.8)		24 (28.6)	0.05	0.11	0.58
Negative	131	55 (75.3)		16 (55.2)		60 (71.4)			
Unknown	1	0		1		0			
ERBB2 status[a]									
Positive	13	3 (4.1)		4 (13.8)		6 (7.1)	0.10	0.28	0.51
Negative	173	70 (95.9)		25 (86.2)		78 (92.9)			
Unknown	1	0		1		0			
Molecular type[a]									
ER positive, PR positive, and ERBB2 negative	58	23 (31.5)		11 (37.9)		24 (28.6)	0.16	0.28	0.69
ER negative, PR negative, and ERBB2 negative	115	47 (64.4)		14 (48.3)		54 (64.3)			
ERBB2 positive	13	3 (4.1)		4 (13.8)		6 (7.1)			
Unknown	1	0		1		0			
Stage[a]									
I	60	31 (42.5)		2 (6.7)		27 (32.9)	<0.001	<0.001	0.46
II	110	40 (54.8)		17 (56.7)		53 (64.6)			
III	15	2 (2.7)		11 (36.7)		2 (2.4)			
Unknown	2	0		0		2			
Grade[a]									

© 2021 Wan Q et al. *JAMA Network Open.*

I+II	104	43 (61.4)		19 (70.4)		42 (57.5)	0.41	0.24	0.64
III	66	27 (38.6)		8 (29.6)		31 (42.5)			
Unknown	17	3		3		11			
Tumor size (cm)[a]									
≤2	74	35 (47.9)		8 (26.7)		31 (38.8)	<0.001	0.001	0.49
(2, 3]	67	24 (32.9)		8 (26.7)		35 (43.8)			
(3, 4]	24	11 (15.1)		4 (13.3)		9 (11.3)			
(4, 5]	6	1 (1.4)		3 (10.0)		2 (2.5)			
>5	12	2 (2.7)		7 (23.3)		3 (3.8)			
Unknown	4	0		0		4			
Nodal status									
0	131	56 (76.7)		7 (23.3)		68 (81.0)	<0.001	<0.001	0.81
1-3	44	16 (21.9)		13 (43.3)		15 (17.9)			
≥4	12	1 (1.4)		10 (33.3)		1 (1.2)			
Adjuvant therapy									
Chemotherapy	115	40 (54.8)		16 (53.3)		59 (70.2)	0.97	0.12	0.02
Endocrine therapy	11	4 (5.5)		2 (6.7)		5 (6.0)			
Chemotherapy and endocrine therapy	58	29 (39.7)		12 (40.0)		17 (20.2)			
None	3	0 (0)		0 (0)		3 (3.6)			

BCT, breast-conserving therapy; RT, radiotherapy; SD, standard deviation; BCOC, breast cancer and/or ovarian cancer; ER, estrogen receptor; PR, progesterone receptor; P^1, BCT vs. mastectomy plus RT; P^2, mastectomy+RT vs. mastectomy alone; P^3, BCT vs. mastectomy alone.
[a]Percentages calculated without unknown numbers included.

© 2021 Wan Q et al. *JAMA Network Open.*

eTable 2. Clinicopathologic Characteristics for *BRCA2* Variant Carriers Receiving Breast-Conserving Therapy, Mastectomy With Radiotherapy, or Mastectomy Alone

**Characteristics**	**No.**	**BCT, N (%)**	**Mastectomy plus RT, N (%)**	**Mastectomy alone, N (%)**	**P[1]**	**P[2]**	**P[3]**
Total	304	106 (34.9)	67 (22.0)	131 (43.1)			
Follow-up(years)							
Mean(±SD)	7.9±3.2	8.5±3.3	7.8±3.3	7.6±3.1	0.22	0.59	0.03
Median	7.3	7.8	6.8	7.1			
Range	0.4-16.0	0.4-15.2	1.8-15.9	1.1-16.0			
Age (years)							
Mean(±SD)	47.6±10.4	44.7±9.1	45.4±9.1	51.0±11.0	0.64	<0.001	<0.001
Median	47	45.5	45	52			
Range	21-75	21-71	29-73	29-75			
≤45	130	53 (50.0)	37 (55.2)	40 (30.5)	0.50	0.001	0.002
>45	174	53 (50.0)	30 (44.8)	91 (69.5)			
Year of diagnosis							
2003-2009	111	41 (38.7)	28 (41.8)	42 (32.1)	0.68	0.18	0.29
2010-2015	193	65 (61.3)	39 (58.2)	89 (67.9)			
Family history of any cancer							
Yes	166	59 (55.7)	35 (52.2)	72 (55.0)	0.66	0.72	0.91
No	138	47 (44.3)	32 (47.8)	59 (45.0)			
Family history of BCOC							
Yes	100	40 (37.7)	17 (25.4)	43 (32.8)	0.09	0.28	0.43
No	204	66 (62.3)	50 (74.6)	88 (67.2)			

© 2021 Wan Q et al. *JAMA Network Open.*

ER status									
Positive	242	90 (84.9)		59 (88.1)		93 (71.0)	0.56	0.007	0.01
Negative	62	16 (15.1)		8 (11.9)		38 (29.0)			
PR status									
Positive	224	85 (80.2)		56 (83.6)		83 (63.4)	0.58	0.003	0.005
Negative	80	21 (19.8)		11 (16.4)		48 (36.6)			
ERBB2 status									
Positive	35	12 (11.3)		7 (10.4)		16 (12.2)	0.86	0.71	0.83
Negative	269	94 (88.7)		60 (89.6)		115 (87.8)			
Molecular type[a]									
ER positive, PR positive, and ERBB2 negative	225	87 (82.1)		53 (79.1)		85 (64.9)	0.66	0.08	0.002
ER negative, PR negative, and ERBB2 negative	44	7 (6.6)		7 (10.4)		30 (22.9)			
ERBB2 positive	35	12 (11.3)		7 (10.4)		16 (12.2)			
Stage[a]									
I	83	34 (32.1)		2 (3.0)		47 (37.0)	<0.001	<0.001	<0.001
II	169	56 (52.8)		34 (51.5)		79 (62.2)			
III	47	16 (15.1)		30 (45.5)		1 (0.8)			
Unknown	5	0		1		4			
Grade[a]									
I+II	221	80 (87.0)		50 (87.7)		91 (74.6)	0.89	0.05	0.03
III	50	12 (13.0)		7 (12.3)		31 (25.4)			
Unknown	33	14		10		9			
Tumor size (cm)[a]									
≤2	137	51 (48.1)		21 (31.8)		65 (50.4)	0.001	0.002	0.94
(2,3]	93	36 (34.0)		16 (24.2)		41 (31.8)			

© 2021 Wan Q et al. *JAMA Network Open.*

(3, 4]	36	13 (12.3)		10 (15.2)		13 (10.1)			
(4, 5]	18	3 (2.8)		10 (15.2)		5 (3.9)			
>5	17	3 (2.8)		9 (13.6)		5 (3.9)			
Unknown	3	0		1		2			
Nodal status[a]									
0	174	68 (64.2)		10 (15.2)		96 (74.4)	<0.001	<0.001	<0.001
1-3	82	22 (20.8)		27 (40.9)		33 (25.6)			
≥4	45	16 (15.1)		29 (43.9)		0 (0.0)			
Unknown	3	0		1		2			
Adjuvant therapy									
Chemotherapy	60	15 (14.2)		8 (11.9)		37 (28.2)	0.01	<0.001	0.007
Endocrine therapy	59	23 (21.7)		4 (6.0)		32 (24.4)			
Chemotherapy and endocrine therapy	179	67 (63.2)		55 (82.1)		57 (43.5)			
None	6	1 (0.9)		0 (0)		5 (3.8)			

BCT, breast-conserving therapy; RT, radiotherapy; SD, standard deviation; BCOC, breast cancer and/or ovarian cancer; ER, estrogen receptor; PR, progesterone receptor; P^1, BCT vs. mastectomy plus RT; P^2, mastectomy plus RT vs. mastectomy alone; P^3, BCT vs. mastectomy alone.
[a]Percentages calculated without unknown numbers included.

© 2021 Wan Q et al. *JAMA Network Open.*

eTable 3. Clinicopathologic Characteristics for Noncarriers Receiving Breast-Conserving Therapy, Mastectomy With Radiotherapy, or Mastectomy Alone

**Characteristics**	**No.**	**BCT, N (%)**	**Mastectomy plus RT, N (%)**	**Mastectomy alone, N (%)**	**P[1]**	**P[2]**	**P[3]**
Total	7905	2956 (37.4)	1414 (17.9)	3535 (44.7)			
Follow-up(years)							
Mean(±SD)	8.1±3.2	8.5±3.3	7.4±3.3	8.0±3.1	<0.001	<0.001	<0.001
Median	7.5	8.1	7.1	7.4			
Range	0.3-16.6	0.3-16.3	0.4-15.6	0.3-16.6			
Age (years)							
Mean(±SD)	51.0±11.4	48.8±11.2	49.4±10.4	53.6±11.5	0.08	<0.001	<0.001
Median	50	48	49	53			
Range	19-90	20-87	19-82	23-90			
≤45	2673	1237 (41.8)	507 (35.9)	929 (26.3)	<0.001	<0.001	<0.001
>45	5232	1719 (58.2)	907 (64.1)	2606 (73.7)			
Year of diagnosis							
2003-2009	3282	1374 (46.5)	563 (39.8)	1345 (38.0)	<0.001	0.25	<0.001
2010-2015	4623	1582 (53.5)	851 (60.2)	2190 (62.0)			
Family history of any cancer							
Yes	2354	933 (31.6)	391 (27.7)	1030 (29.1)	0.008	0.30	0.03
No	5551	2023 (68.4)	1023 (72.3)	2505 (70.9)			
Family history of BCOC							
Yes	628	249 (8.4)	103 (7.3)	276 (7.8)	0.20	0.53	0.37
No	7277	2707 (91.6)	1311 (92.7)	3259 (92.2)			

© 2021 Wan Q et al. *JAMA Network Open.*

ER status[a]									
Positive	5770	2233 (75.7)		1019 (72.2)		2518 (71.6)	0.01	0.67	<0.001
Negative	2106	716 (24.3)		392 (27.8)		998 (28.4)			
Unknown	29	7		3		19			
PR status[a]									
Positive	5196	2078 (70.5)		900 (63.8)		2218 (63.1)	<0.001	0.65	<0.001
Negative	2675	870 (29.5)		510 (36.2)		1295 (36.9)			
Unknown	34	8		4		22			
ERBB2 status[a]									
Positive	1938	608 (20.6)		408 (28.9)		922 (26.3)	<0.001	0.06	<0.001
Negative	5927	2337 (79.4)		1003 (71.1)		2587 (73.7)			
Unknown	40	11		3		26			
Molecular type[a]									
ER positive, PR positive, and ERBB2 negative	4914	1950 (66.2)		824 (58.4)		2140 (61.0)	<0.001	0.16	<0.001
ER negative, PR negative, and ERBB2 negative	1012	386 (13.1)		179 (12.7)		447 (12.7)			
ERBB2 positive	1938	608 (20.7)		408 (28.9)		922 (26.3)			
Unknown	41	12		3		26			
Stage[a]									
I	2347	1125 (39.3)		32 (2.3)		1190 (34.6)	<0.001	<0.001	<0.001
II	4385	1533 (54.3)		671 (47.8)		2161 (62.9)			
III	965	181 (6.3)		700 (49.9)		85 (2.4)			
Unknown	208	97		11		100			
Grade[a]									
I+II	5845	2247 (85.4)		1100 (85.9)		2498 (83.7)	0.68	0.07	0.08
III	1049	383 (14.6)		180 (14.1)		486 (16.3)			

© 2021 Wan Q et al. *JAMA Network Open.*

Unknown	1011	326		134		551			
Tumor size (cm) [a]									
≤2	3436	1540 (53.3)		338 (24.2)		1558 (45.3)	<0.001	<0.001	<0.001
(2, 3]	2466	948 (32.8)		401 (28.7)		1117 (32.5)			
(3, 4]	1150	301 (10.4)		319 (22.8)		530 (15.4)			
(4, 5]	310	61 (2.1)		123 (8.8)		126 (3.7)			
>5	364	38 (1.3)		216 (15.5)		110 (3.2)			
Unknown	179	68		17		94			
Nodal status[a]									
0	4895	2047 (70.3)		215 (15.2)		2633 (75.0)	<0.001	<0.001	<0.001
1-3	2056	693 (23.8)		551 (39.1)		812 (23.1)			
≥4	880	172 (5.9)		644 (45.7)		64 (1.8)			
Unknown	74	44		4		26			
Adjuvant therapy									
Chemotherapy	1797	608 (20.6)		359 (25.4)		830 (23.5)	<0.001	<0.001	<0.001
Endocrine therapy	1980	874 (29.6)		52 (3.7)		1054 (29.8)			
Chemotherapy and endocrine therapy	3836	1413 (47.8)		996 (70.4)		1427 (40.4)			
None	292	61 (2.1)		7 (0.5)		224 (6.3)			

BCT, breast-conserving therapy; RT, radiotherapy; SD, standard deviation; BCOC, breast cancer and/or ovarian cancer; ER, estrogen receptor; PR, progesterone receptor; C, chemotherapy; E, endocrine therapy; P^1, BCT vs. mastectomy plus RT; P^2, mastectomy plus RT vs. mastectomy alone; P^3, BCT vs. mastectomy alone.
[a]Percentages calculated without unknown numbers included.

© 2021 Wan Q et al. *JAMA Network Open.*

eTable 4. Comparison of Survival Between Breast-Conserving Therapy, Mastectomy With Radiotherapy, and Mastectomy Alone in *BRCA1* and *BRCA2* Variant Carriers, and Noncarriers Among the Patients with Early-Stage Disease (T3N0M0 Excluded)

Survival	Surgery	Univariate analysis			Multivariable analysis[a]	
		HR (95% CI)	*P*		HR (95% CI)	*P*
BRCA1						
RFS	BCT vs. Mastectomy plus RT	0.55(0.17-1.72)	0.30		0.64 (0.17-2.44)	0.52
	BCT vs. Mastectomy alone	0.90(0.40-2.06)	0.81		1.24 (0.50-3.11)	0.64
DRFS	BCT vs. Mastectomy plus RT	0.49(0.15-1.56)	0.23		0.63 (0.15-2.63)	0.53
	BCT vs. Mastectomy alone	1.16(0.47-2.83)	0.75		1.54 (0.57-4.15)	0.39
BCSS	BCT vs. Mastectomy plus RT	1.29(0.15-10.71)	0.82		1.31 (0.12-14.49)	0.83
	BCT vs. Mastectomy alone	0.86(0.27-2.72)	0.79		1.12 (0.30-4.22)	0.87
OS	BCT vs. Mastectomy plus RT	1.48(0.18-12.08)	0.71		1.31 (0.13-13.33)	0.82
	BCT vs. Mastectomy alone	0.92(0.32-2.62)	0.87		1.29 (0.39-4.24)	0.68
BRCA2						
RFS	BCT vs. Mastectomy plus RT	0.40(0.16-0.98)	0.05		0.66 (0.23-1.90)	0.44
	BCT vs. Mastectomy alone	0.81(0.37-1.78)	0.59		0.73 (0.32-1.67)	0.46
DRFS	BCT vs. Mastectomy plus RT	0.36(0.14-0.97)	0.04		0.58 (0.18-1.86)	0.36
	BCT vs. Mastectomy alone	0.86(0.35-2.10)	0.73		0.85 (0.33-2.16)	0.73
BCSS	BCT vs. Mastectomy plus RT	0.34(0.09-1.37)	0.13		0.76 (0.13-4.50)	0.77
	BCT vs. Mastectomy alone	0.97(0.26-3.64)	0.97		0.90 (0.23-3.60)	0.89
OS	BCT vs. Mastectomy plus RT	0.34(0.09-1.37)	0.13		0.98 (0.18-5.21)	0.98
	BCT vs. Mastectomy alone	0.70(0.20-2.38)	0.56		0.60 (0.16-2.20)	0.44

© 2021 Wan Q et al. *JAMA Network Open.*

Non-carriers						
RFS	BCT vs. Mastectomy plus RT	0.56(0.45-0.70)	<0.001		0.89 (0.70-1.12)	0.32
	BCT vs. Mastectomy alone	0.84(0.72-0.97)	0.02		0.82 (0.71-0.96)	0.01
DRFS	BCT vs. Mastectomy plus RT	0.43(0.34-0.54)	<0.001		0.67 (0.52-0.86)	0.002
	BCT vs. Mastectomy alone	0.72(0.61-0.86)	<0.001		0.73 (0.61-0.86)	<0.001
BCSS	BCT vs. Mastectomy plus RT	0.34(0.25-0.46)	<0.001		0.56 (0.40-0.79)	0.001
	BCT vs. Mastectomy alone	0.70(0.54-0.90)	0.005		0.69 (0.54-0.90)	0.006
OS	BCT vs. Mastectomy plus RT	0.39(0.29-0.52)	<0.001		0.58 (0.43-0.80)	0.001
	BCT vs. Mastectomy alone	0.64(0.51-0.79)	<0.001		0.68 (0.54-0.85)	0.001

BCT, breast-conserving therapy; RT, radiotherapy; RFS, recurrence-free survival; DRFS, distant recurrence-free survival; BCSS, breast cancer-specific survival; OS, overall survival; 95% CI, 95% confidence interval.

[a] Hazard ratio adjusted clinicopathologic characteristics and treatment factors including age at breast cancer diagnosis (≤45 vs. >45 years), family history of breast cancer and/or ovarian cancer (with vs. without), years of diagnosis (2003-2009 vs. 2010-2015), estrogen receptor status, progesterone receptor status, ERBB2 status, lymph node status (Positive vs. Negative), tumor size (>3 vs. ≤3 cm), grade (III vs. I+II, Unknown vs. I and II) and adjuvant therapy (chemotherapy vs. endocrine therapy and no therapy).

© 2021 Wan Q et al. *JAMA Network Open.*

eTable 5. Risk of Ipsilateral Breast Tumor Recurrence for Patients Receiving Different Type of Surgery Within *BRCA1* and *BRCA2* Variant Carriers, and Noncarriers

**Surgery**	**No.**	**IBTR**		**OR (95% CI)**	***P***
		**Events**	**%**		
BRCA1 carriers					
BCT	73	1	1.4	0.38(0.04-3.69)	0.62
Mastectomy plus RT	30	0	0.0	-	0.57
Mastectomy alone	84	3	3.6	1.0(Ref)	-
Total	187	4	2.1		
BRCA2 carriers					
BCT	106	8	7.5	2.06(0.65-6.49)	0.21
Mastectomy plus RT	67	1	1.5	0.38(0.04-3.34)	0.67
Mastectomy alone	131	5	3.8	1.0(Ref)	-
Total	304	14	4.6		
Non-carriers					
BCT	2956	115	3.9	1.45(1.10-1.91)	0.008
Mastectomy plus RT	1414	38	2.7	0.99(0.68-1.45)	0.96
Mastectomy alone	3535	96	2.7	1.0(Ref)	-
Total	7905	249	3.1		

BCT, breast-conserving therapy; RT, radiotherapy; IBTR, ipsilateral breast tumor recurrence; OR, odds ratios; 95% CI, 95% confidence interval.

© 2021 Wan Q et al. *JAMA Network Open.*

eTable 6. Risk of Ipsilateral Breast Tumor Recurrence for *BRCA1* and *BRCA2* Variant Carriers, and Noncarriers in Subgroups of Different Type of Surgery

Surgery	No.	IBTR		OR (95% CI)	*P*
		Events	%		
BCT					
BRCA1	73	1	1.4	0.34(0.05-2.49)	0.53
BRCA2	106	8	7.5	2.02(0.96-4.25)	0.07
Non-carriers	2956	115	3.9	1.0(Ref)	-
Total	3135	124	4.0		
Mastectomy plus RT					
BRCA1	30	0	0.0	-	1.0
BRCA2	67	1	1.5	0.55(0.07-4.06)	1.0
Non-carriers	1414	38	2.7	1.0(Ref)	-
Total	1511	39	2.6		
Mastectomy alone					
BRCA1	84	3	3.6	1.33(0.41-4.28)	0.50
BRCA2	131	5	3.8	1.42(0.57-3.55)	0.41
Non-carriers	3535	96	2.7	1.0(Ref)	-
Total	3750	104	2.8		

BCT, breast-conserving therapy; RT, radiotherapy; IBTR, ipsilateral breast tumor recurrence; OR, odds ratios; 95% CI, 95% confidence interval.

© 2021 Wan Q et al. *JAMA Network Open.*

eTable 7. Risk of Contralateral Breast Cancer for Patients Receiving Different Type of Surgery Within *BRCA1* and *BRCA2* Variant Carriers, and Noncarriers

Surgery	No.	CBC		OR (95% CI)	*P*
		Events	%		
BRCA1 carriers					
BCT	73	10	13.7	1.18(0.46-3.00)	0.74
Mastectomy plus RT	30	6	20.0	1.85(0.61-5.62)	0.36
Mastectomy alone	84	10	11.9	1.0(Ref)	-
Total	187	26	13.9		
BRCA2 carriers					
BCT	106	7	6.6	0.29(0.12-0.69)	0.003
Mastectomy plus RT	67	8	11.9	0.55(0.23-1.29)	0.16
Mastectomy alone	131	26	19.8	1.0(Ref)	-
Total	304	41	13.5		
Non-carriers					
BCT	2956	38	1.3	0.36(0.25-0.52)	<0.001
Mastectomy plus RT	1414	36	2.5	0.72(0.49-1.05)	0.08
Mastectomy alone	3535	124	3.5	1.0(Ref)	-
Total	7905	198	2.5		

BCT, breast-conserving therapy; RT, radiotherapy; CBC, contralateral breast cancer; OR, odds ratios; 95% CI, 95% confidence interval.

© 2021 Wan Q et al. *JAMA Network Open.*

eFigure 1. Flow Diagram of the Patients Included in This Cohort
BCT, breast-conserving therapy; RT, radiotherapy.

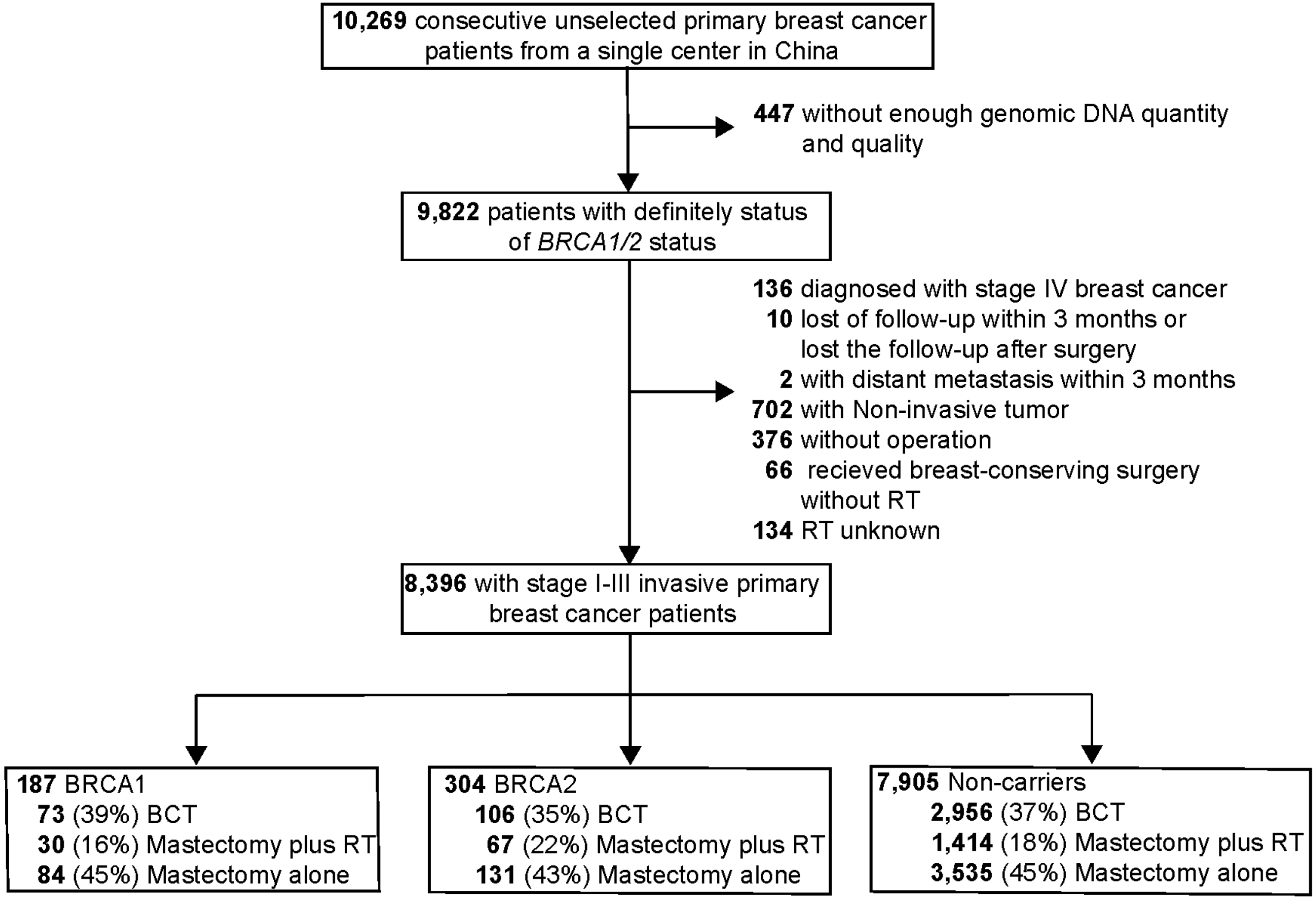

© 2021 Wan Q et al. *JAMA Network Open.*

eFigure 2. Survival between *BRCA1* and *BRCA2* Variant Carriers, and Noncarriers in This Cohort
A), Recurrence-free survival; B) Distant recurrence-free survival; C), breast cancer-specific survival; D), Overall survival.

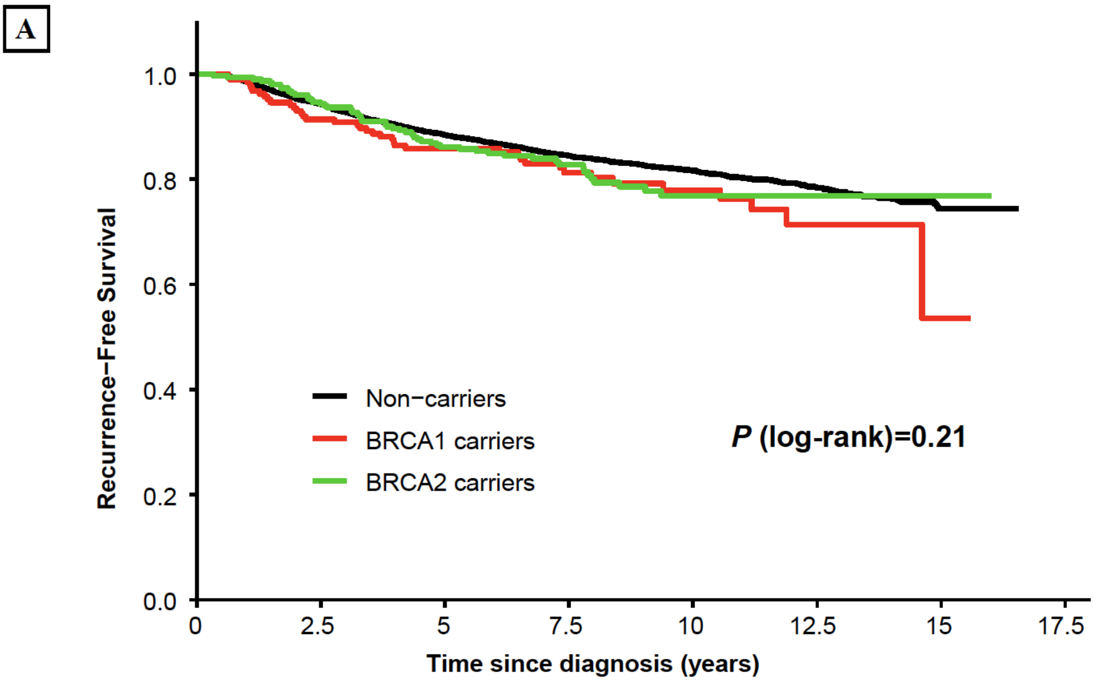

No. at risk

Non−carriers	7905	7377	6401	3665	2168	710	165	0
BRCA1	187	168	146	93	58	16	3	0
BRCA2	304	282	235	131	74	34	7	0

© 2021 Wan Q et al. *JAMA Network Open.*

B

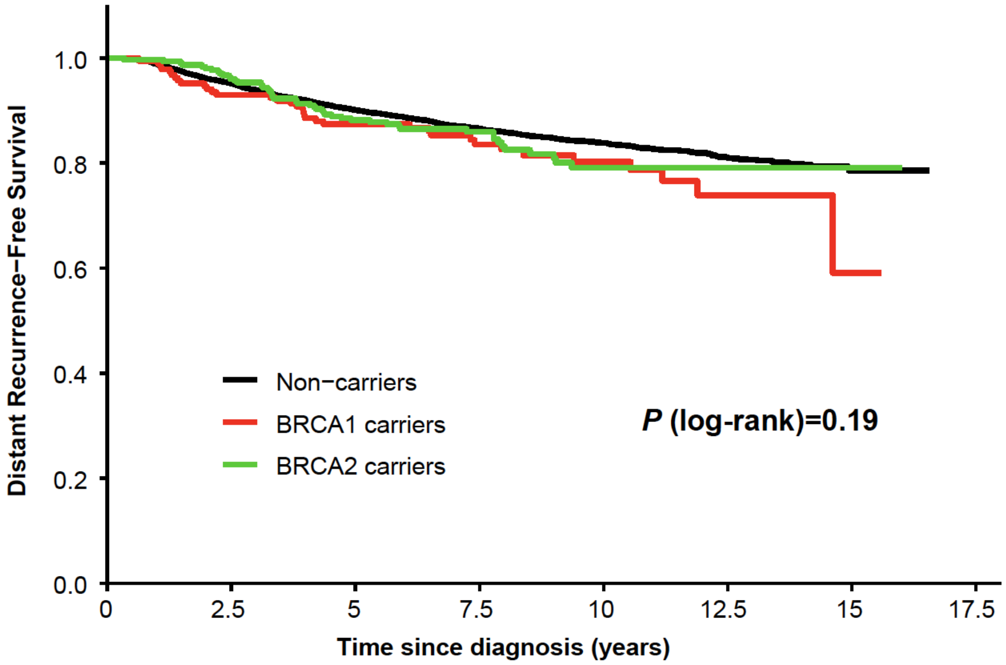

No. at risk

Non−carriers	7905	7443	6523	3767	2235	752	181	0
BRCA1	187	170	148	96	61	18	4	0
BRCA2	304	287	241	135	75	34	7	0

© 2021 Wan Q et al. *JAMA Network Open.*

C

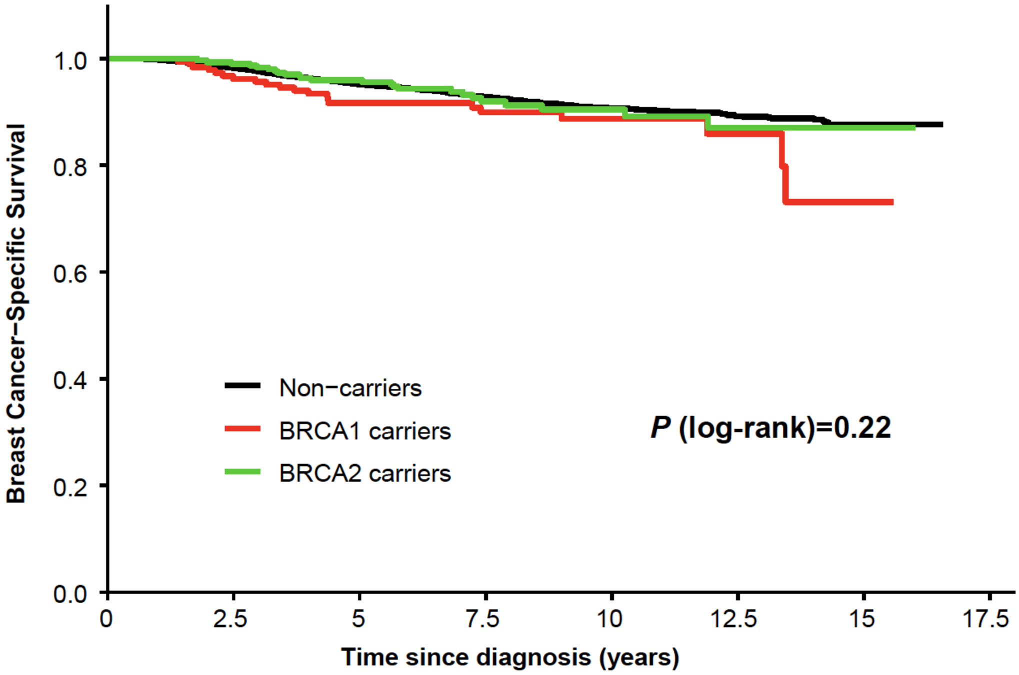

No. at risk

Non−carriers	7905	7637	6799	3974	2343	805	192	0
BRCA1	187	176	151	100	64	22	5	0
BRCA2	304	296	260	141	84	37	9	0

© 2021 Wan Q et al. *JAMA Network Open.*

D

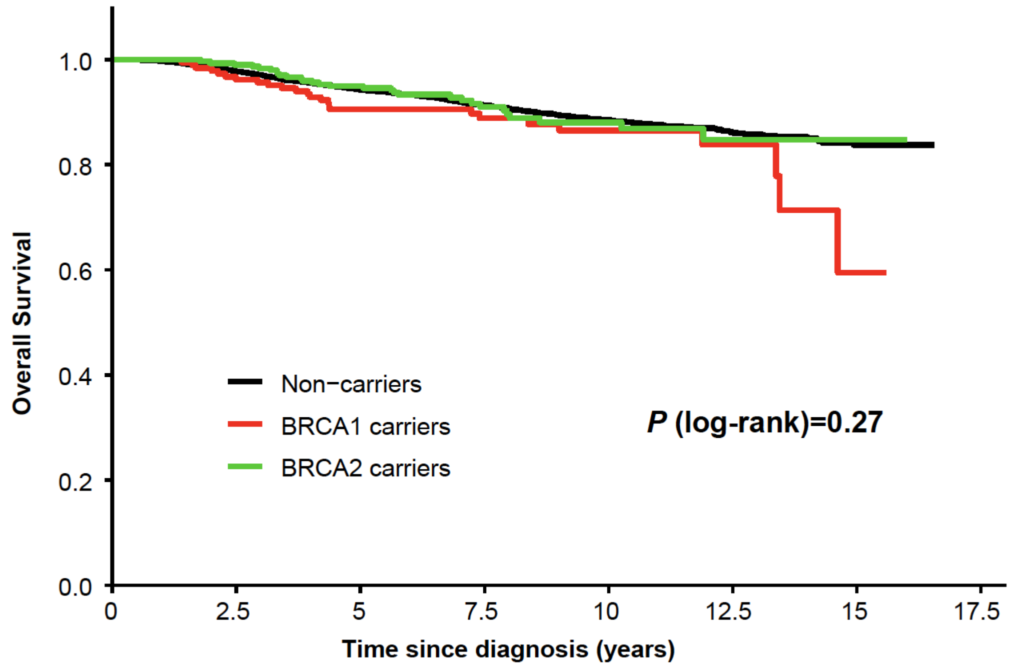

No. at risk

Non−carriers	7905	7637	6799	3974	2343	805	192	0
BRCA1	187	176	151	100	64	22	5	0
BRCA2	304	296	260	141	84	37	9	0

© 2021 Wan Q et al. *JAMA Network Open.*
